# Supplementary material for: What are the probable predictors of urinary incontinence during pregnancy?
Source: PeerJ. 2016 Jul 27;4:e2283. doi: 10.7717/peerj.2283 (PMC4974920; doi:10.7717/peerj.2283)
Supplement: Supplemental Information 1 [file peerj-04-2283-s002.doc]

Supplement1-**ICIQ-SF** ^ɸ^

**CONFIDENTIAL**

Many people have urinary leakage at times. We are trying to find out the number of people with urinary leakage and how much of a bother this is to them. We would be grateful if you could answer the questions below considering how you were on average within the last month.

1. How often do you have urinary leakage? Never 0

About once a week or less 1

Twice or three times a week 2

About once a day 3

Few times a day 4

Always 5

1. We would like to know how much urinary leakage you think you have. How much urinary leakage do you have in general (regardless you wear pads or not)?

None 0

A small amount 2

A moderate amount 4

A large amount 6

1. Overall , how much does urinary leakage affect your daily life?

Please circle a number from 0 (not at all) to 10 (to a great extent)

0 1 2 3 4 5 6 7 8 9 10

Not at all To a great extent

(ICIQ-SF Score=1+2+3)

1. When does urine leake? (Please mark all that apply to you)

(never) urine does not leak

leaks before you reach the toilet

leaks when you cough or sneeze

leaks while you are sleeping

physically active or exercising

leaks when you have finished urinating and get dressed up

leak without an apparent reason

leaks all the time

5 – Please mark life style changes because of urinary leakage (all that apply to you)

Affect shopping or excursions outside the home

Affect working performance and friendship

Affect daily home activities

Affect general health status

Affect sexual relations

Makes you nervous and anxious

Need wearing pad or protector
