# Supplementary material for: What are the probable predictors of urinary incontinence during pregnancy?
Source: PeerJ. 2016 Jul 27;4:e2283. doi: 10.7717/peerj.2283 (PMC4974920; doi:10.7717/peerj.2283)
Supplement: Supplemental Information 2 [file peerj-04-2283-s003.doc]

Supplement 2 - QOL-Questionnaire of The Patients with Urinary **^µ^** Incontinence:

Following each sentence is related to urinary incontinence, in which case the person's life is meant to measure how much influenced.

Please encode every sentence as follows: 0: No, 1: Mild, 2: Moderate, 3: Very

1.I wonder if I soak my clothes, so I am constantly worried.

2. While talking this issue to others, I'm so ashamed, I feel embarrassed.

3. I constantly have to pay attention to the amount of liquid I received.

4. I wonder if I cough or sneeze, so I am always anxious.

5. I need to be very careful while getting up after I sit down.

6. I wonder where the toilet is when I go to a new place.

7. I feel unhappy.

8. I do not want to leave my house for a long time if I pass urine.

9. My self-confidence gets damaged because of that.

10. I am frustrated because I can not do anything I want.

11 I'm always afraid if others will smell the odor of urine on me.

12. Urinary incontinence constantly stays in my mind.

13. It is very important for me to go toilet very often.

14. I avoid laughter.

15. I am in a constant embarrassment because of incontinence.

16. I need to plan every further detail related to urinary incontinence.

17. I worried I would be worse as the older I'm.

18. I find it hard to have a good sleep at night.

19. Every moment I wonder if something will break my pride, or I am concerned about if I will suffer from urinary incotinence.

20. I avoid from embracing other people.

21. Urinary incontinence is giving the impression that I am not a healthy man.

22. It makes me feel desperate.

23. I enjoy life less.

24. I'm afraid I will not reach the toilet in time.

25. I have the feeling I can not control the bladder.

26. I need to be in a constant attention.

27. This limits my choice of clothing.

28. I'm worried if my sex life would be affected.
